# Haptic size perception is influenced by body and object orientation

**DOI:** 10.1038/s41598-025-95800-6

**Published:** 2025-04-23

**Authors:** M. McManus, L. R. Harris, K. Fiehler

**Affiliations:** 1 Justus Liebig University Giessen, Giessen, Germany; 2 Centre for Vision Research, York University, Toronto, ON Canada

**Keywords:** Haptic size perception, Vestibular, Multisensory integration, Body tilt, Spatial representation, Gravity, Posture, Psychology, Human behaviour

## Abstract

Changes in body orientation from standing have been shown to impact our perception of visual size. This has been attributed to the vestibular system’s involvement in constructing a representation of the space around our body. In the current study we investigated how body posture influences haptic size perception. Blindfolded participants were tasked with estimating the felt length of a rod and then adjusting it back to its previously felt size (after it had been set to a random length). Participants could feel and adjust the rod in the same posture, standing or supine, or after a change in posture. If the body orientation relative to gravity impacts size perception, we might expect changes in haptic size perception following body tilt. In support of this hypothesis, after changing between standing and supine postures there was a change in the rod’s haptically perceived length but only when the orientation of the rod itself also changed with respect to gravity but not when its orientation was constant. This suggests that body posture influences not only visual but also haptic size perception, potentially due to the vestibular system contributing to the encoding of space with respect to gravity.

## Introduction

Most of the tasks we complete throughout the day, from walking to reaching, are done in an upright posture. Posture has been shown to affect the perceived visual size of objects^1–3^ but might it also affect haptic size perception? Studies on visual size perception have generally used a fixed-length physical reference rod to which participants match the length of an adjustable visual line. Participants typically set the line 11% longer^2^ or 5.4–10.1% longer^3^ when estimates are made while supine compared to when standing. Setting the visual line as too long could be due to an error in the perceived length of the line or in the perceived distance to the line, or both. But there is another potential contributor to this perceptual error: the perceived haptic size of the physical reference rod itself. That at least some of the effect may be visual (either changes in perceived visual size or perceived distance) is indicated by changes in perceived visual size found in the microgravity of space without the use of a tactile reference^4^. However, based on previous results in microgravity^4,5^, it is conceivable that haptic size estimation may also be affected by exposure to microgravity and explain the expansion of haptic size found upon return to 1 g^5^. Before exposure to microgravity, cosmonauts tended to haptically perceive a circle as an ellipse, but after exposure to microgravity, their haptic judgements became more accurate^5^. Relating this back to the previous studies on earth^2,3^, it is possible that the physical comparison rod used as a reference in these visual size judgements could have been haptically misperceived as larger when the participant and rod were tilted together in addition to any visual distortions that led the participant to set the visual line as larger. Why might changes in posture lead to changes in size perception, either visual or haptic? While the exact reason is not currently known, it could be due to misinterpreting vestibular cues^6^ or to changes in the reliability of the vestibular cues with tilt possibly due to differences in the number of hair cells in between utricle and saccule^7,8^ and different firing sensitivities^9^ which might lead to tilt-dependant noise^10^, see also^11,12^ for behavioural studies that show lower precision of estimates while tilted. A change in the reliability of a sensory cue with body tilt might lead to changes in the precision of estimates using that cue, with a decrease in precision expected when the signal was least reliable^13,14^. Changes in precision might then affect sensory integration leading to changes in perceived size with body tilt^15,16^. Participants might also show a general bias when adjusting a rod back to a previously felt length that they had been asked to remember^17^ even without changes in posture. Previous studies in delayed grasping have found that when people reach to an unseen object after a 5 s delay there is an increase in their grip aperture suggesting the object’s size might be misremembered as being larger than it was^17^. If these were the only driving factors, then any change in the perceived size of a rod should be found regardless of the orientation in which it was held.

The vestibular system has been suggested to be involved in the construction of the representation of the space around us^18,19^ and might help to interpret other sensory information or create frames of reference^2,19^. When tilted or in microgravity, this representation may become distorted^18,20,21^. Even while standing upright, the perception of the space around us is non-isotropic, that is, it is distorted differently in different directions. In particular, our visual perception of depth is scaled differently horizontally and vertically^22–24^. Ascertaining whether such distortions are also found in haptic space is one aim of the present study. If a rod were to be held aligned with the long axis of the body and then the person holding the rod were tilted, the rod’s orientation in space relative to gravity would be changed. In contrast, if the rod were held across the body, its orientation relative to gravity would remain constant. Any changes in perceived haptic length dependent on rod orientation could thus not be due to changes in the reliability of the vestibular cue (which would affect all estimates of size), but instead due to the way in which the vestibular system contributes to encoding space with respect to gravity. This would also explain the unusual results in Harris and Mander^2^ where participants show a similar compression in the perceived size of the visual line while supine and also while made to feel supine in a tilted room while they were actually upright as participants in that condition would be misperceiving where gravity is.

### Current study

In the current study we investigated if changes in whole body tilt can influence the perceived haptic size of objects. Blindfolded participants haptically estimated the length of a rod (the “sample” phase) while holding the rod either longitudinally (along the length of their body, where its orientation changed relative to gravity when participants were tilted) or laterally (across their chest, where it didn’t) in a standing or supine posture. The rod was then removed and the participants either changed their posture or continued to stay in that posture. Afterwards the rod was given back but was set to a random size. Their task was then to adjust the rod to its remembered initial length (the “setting” phase). If the results of the Kim et al.^3^, and Harris and Mander^2^ were at least partially due to a haptic expansion during body tilt we would expect that when our participants were supine they would misperceive the length of the rod as longer than it really was. One important caveat is that in order to determine if changes in posture impact haptic size perception the participants have to change their posture between sampling and setting the rod. When maintaining a posture, we can only measure general biases in setting the length of the rod.

Any posture-related differences independent of rod orientation would support the idea that the previous results^2,3^ were exclusively due to changes in the reliability of the vestibular cue with body tilt, while any differences dependent on rod orientation would support the idea that the results were at least partially due to the way in which the vestibular system encodes space relative to gravity.

**Fig. 1:**
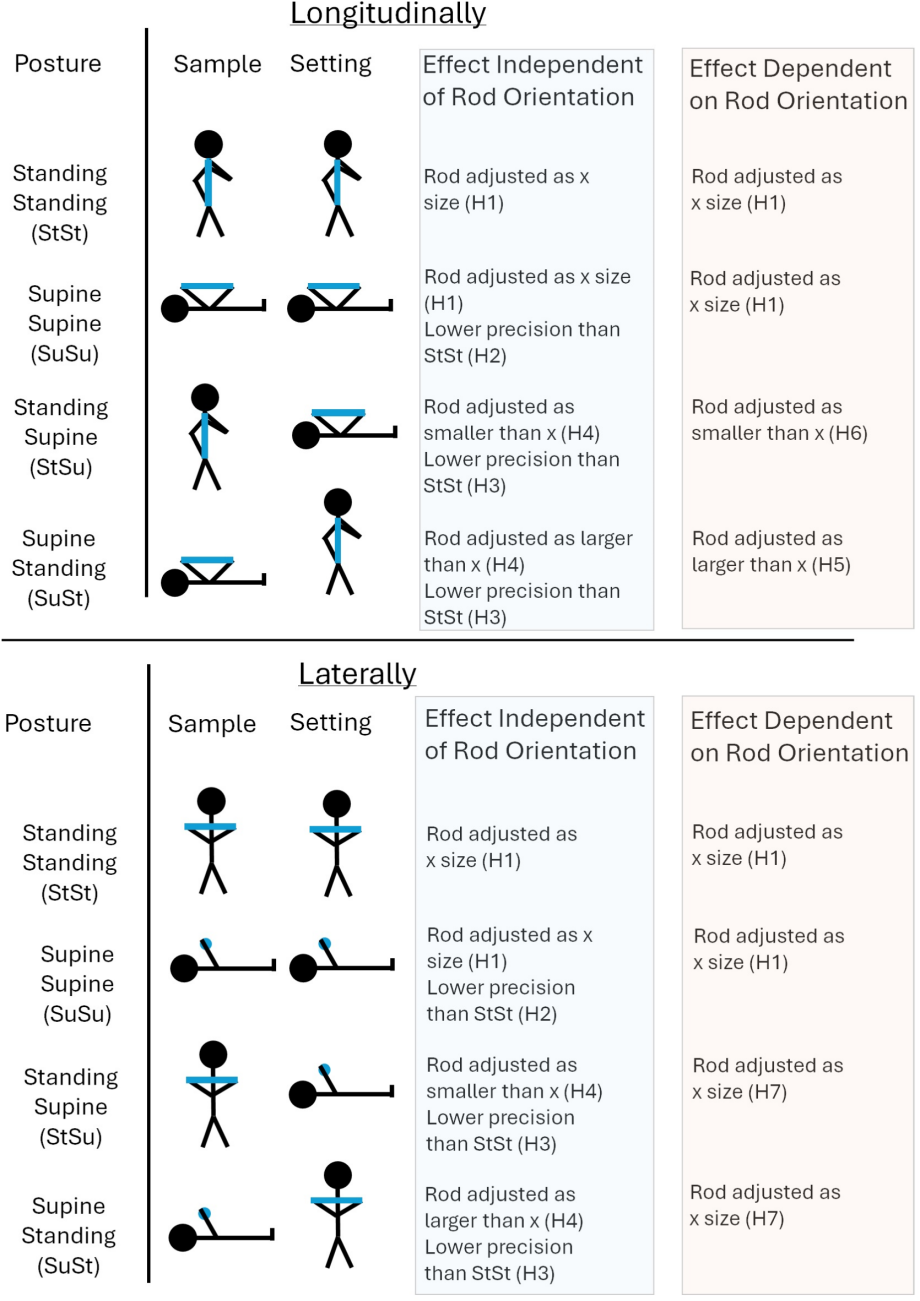
A depiction of the possible results depending on if any effects seen are dependant or independent of rod orientation. The top panel provides the effects if the rod is held longitudinally and the bottom panel if the rod is held laterally. The posture column provides the names of the different posture combinations. The sample and setting columns depict the posture the participant will be in as well as how the rod will be held. The possible effects based on if the results are dependant or independent of rod orientation are then described in the blue and orange columns respectively. “X” refers to the size the participant will set the rod when they maintain their posture. The hypotheses are labelled as “H” followed by the number of the hypothesis.

Our seven hypotheses are outlined in Fig. 1.

### Hypotheses

#### General

##### Hypothesis 1

There will be a bias in the adjusted size of the rod in the setting phase relative to the sampling phase regardless of changes in posture in which the rod will have to be made larger to match the remembered size.

#### Haptic size estimation independent of rod orientation

##### Hypothesis 2

When participants adjust the length of a rod without changing posture between the sample and the setting phase, they will be less precise in the supine posture than in the standing posture.

##### Hypothesis 3

Participants will be less precise in their setting if they change posture between sample and setting than if they don’t.

##### Hypothesis 4

When changing between postures there will be further biases in the adjusted size of the rod. These biases will differ between when participants change from standing to supine (due to a decrease in the reliability of the vestibular cue) and supine to standing (due to an increase in the reliability of the vestibular cue). These differences will reflect a bias towards perceiving the rod as longer than it is while supine. When participants move from a supine posture in the sample phase to a standing posture in the setting phase, a rod of the same length will feel smaller than it was when supine so participants will adjust the rod to be larger to compensate. Conversely, when participants are standing during the sample phase and then lie supine, a rod of the same length will feel larger while supine, so they will adjust the rod smaller to compensate. This will be irrespective of the orientation of the rod (longitudinal vs. lateral).

See Fig. 1 for a diagram of the hypotheses.

#### Haptic Size estimation dependant on rod orientation

##### Hypothesis 5

When holding the rod longitudinally, when participants are supine during the sample phase the rod will feel larger than it really is. Then when they stand upright during the setting phase, a rod of the same length will feel smaller than when supine, so participants will adjust the rod to be larger to compensate.

##### Hypothesis 6

When holding the rod longitudinally, when participants are standing during the sample phase and then lie supine in the setting phase, a rod of the same length will feel larger while supine, so they will adjust the rod smaller to compensate.

##### Hypothesis 7

When holding the rod laterally there will be no affect of changing posture on the perceived length of the rod.

See Fig. 1 for a diagram of the hypotheses.

## Results

In the following sections we describe the results of four t-tests investigating if there is a significant bias in the perceived size of the rod when posture does not change, as well as four repeated measures ANOVAs which were run to determine if changes in body posture affected the perceived length of the rod and the precision of the estimate. The t-tests and ANOVAs were run for each rod orientation separately.

The perceived length of the rod was calculated as the percent difference of the initial and adjusted length of the rod. The average percent error was then calculated for each posture combination and rod orientation for each participant. We refer to this value as the “perceived size difference”.

The precision of the estimate refers to the standard deviation of the perceived size difference within a posture combination for each rod orientation, for each participant. As the standard deviation increases the precision decreases.

A more detailed description of the statistical tests and the calculation of the values used are provided in the “Data Analysis” section.

### Longitudinal rod orientation

#### Measuring bias when the posture was not changed

When participants maintained their posture and held the rod longitudinally there was a significant bias both in the adjusted length of the rod while standing (St–St) *t*(21) = 4.44, *p* < 0.001, *d* = 0.95, and while supine (Su–Su) *t*(21) = 5.73, *p* < 0.001, *d* = 1.22. In both cases the rod was set as larger than it really was (St–St: mean perceived size difference = 3.66%, SD = 3.86%; Su–Su: mean perceived size difference = 3.50%, SD = 2.87%).

#### Effect of posture on the perceived length of the rod

**Fig. 2:**
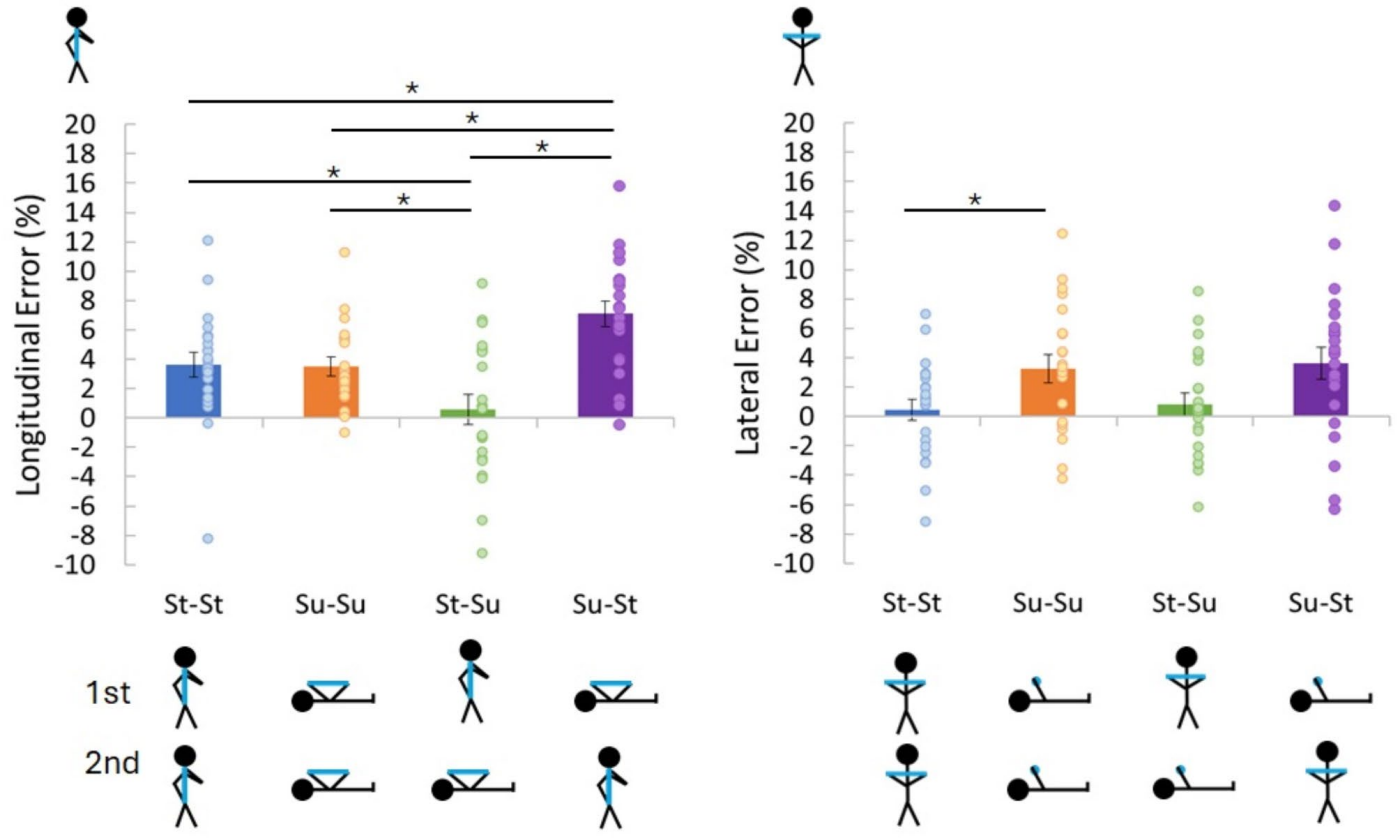
Mean adjusted length of the rod as a percent error of the actual length of the rod (perceived size difference) for all combinations of supine and standing postures. The left panel shows the results for when the rod was held longitudinally and the right panel for when the rod was held laterally. St–St, Standing–Standing; Su–Su, Supine–Supine; St–Su, Standing–Supine; Su–St, Supine–Standing. Error bars are ± standard error. Asterisks indicate significant differences.

For the longitudinal rod orientation, there was a significant effect of body orientation, F(3, 63) = 15.48, *p* < 0.001, n_p_^2^ = 0.42 (Fig. 2; Table 1). Post-hoc comparisons revealed that both the Su–St condition significantly differed from all the other conditions (Su–St > all other conditions) and the St–Su condition from all the other conditions (St–Su < all other conditions). The alpha values for the post-hoc tests have been corrected for the multiple comparisons (Bonferroni) within a given post-hoc t-test such that a ‘corrected alpha’ of less than 0.05 indicates statistical significance. The amount of correction is based on the number of comparisons made. All other post-hoc tests described in the sections below utilized the same method of correction. The mean perceived size differencepercent error in the Su–St condition was 7.10% (SD = 3.98%) while the mean perceived size difference in the St–Su condition was 0.56% (SD = 4.67%). No other effects were found. To confirm the null effects, we calculated the Bayes Factor for any paired samples t-test and provide the results alongside the frequentist results in Table 1.

**Table 1:** The pairwise comparisons for the different rod and body orientations.

Orientation	Condition (I)	Condition (J)	Perceived size difference (I–J)	Standard Error (%)	Corrected alpha	95% confidence interval for difference (%)	Bayes factor 01
Longitudinally	St–St	Su–Su	0.155	0.712	1.000	− 1.920 to 2.230	5.987
	St–St	St–Su	3.098*	1.043	0.044*	0.061 to 6.135	–
	St–St	Su–St	− 3.449*	0.945	0.009*	− 6.202 to (− 0.696)	**–**
	Su–Su	St–Su	2.943*	0.935	0.029*	0.219 to 5.667	–
	Su–Su	Su–St	− 3.604*	0.872	0.003*	− 6.143 to (− 1.064)	**–**
	St–Su	Su–St	− 6.547*	1.198	< 0.001*	− 10.035 to (− 3.059)	**–**
Laterally	St–St	Su–Su	− 2.800	0.839	0.019*	− 5.242 to (− 0.358)	**–**
	St–St	St–Su	− 0.341	0.836	1.000	− 2.775 to 2.092	5.652
	St–St	Su–St	− 3.179	1.101	0.053	− 6.386 to 0.028	0.206
	Su–Su	St–Su	2.459	0.957	0.107	− 0.329 to 5.247	0.383
	Su–Su	Su–St	− 0.379	0.999	1.000	− 3.288 to 2.531	5.715
	St–Su	Su–St	− 2.838	1.394	0.328	− 6.897 to 1.222	0.983

Asterisks indicate significant results. The alpha values have been corrected for the multiple comparisons (Bonferroni) such that a ‘corrected alpha’ of less than 0.05 indicates statistical significance. A Bayes factor (BF 01) above 1 indicates support for the null hypothesis and numbers below 1 indicate support for the test hypothesis.

#### Effect of posture on the precision of the perceived length of the rod

**Fig. 3:**
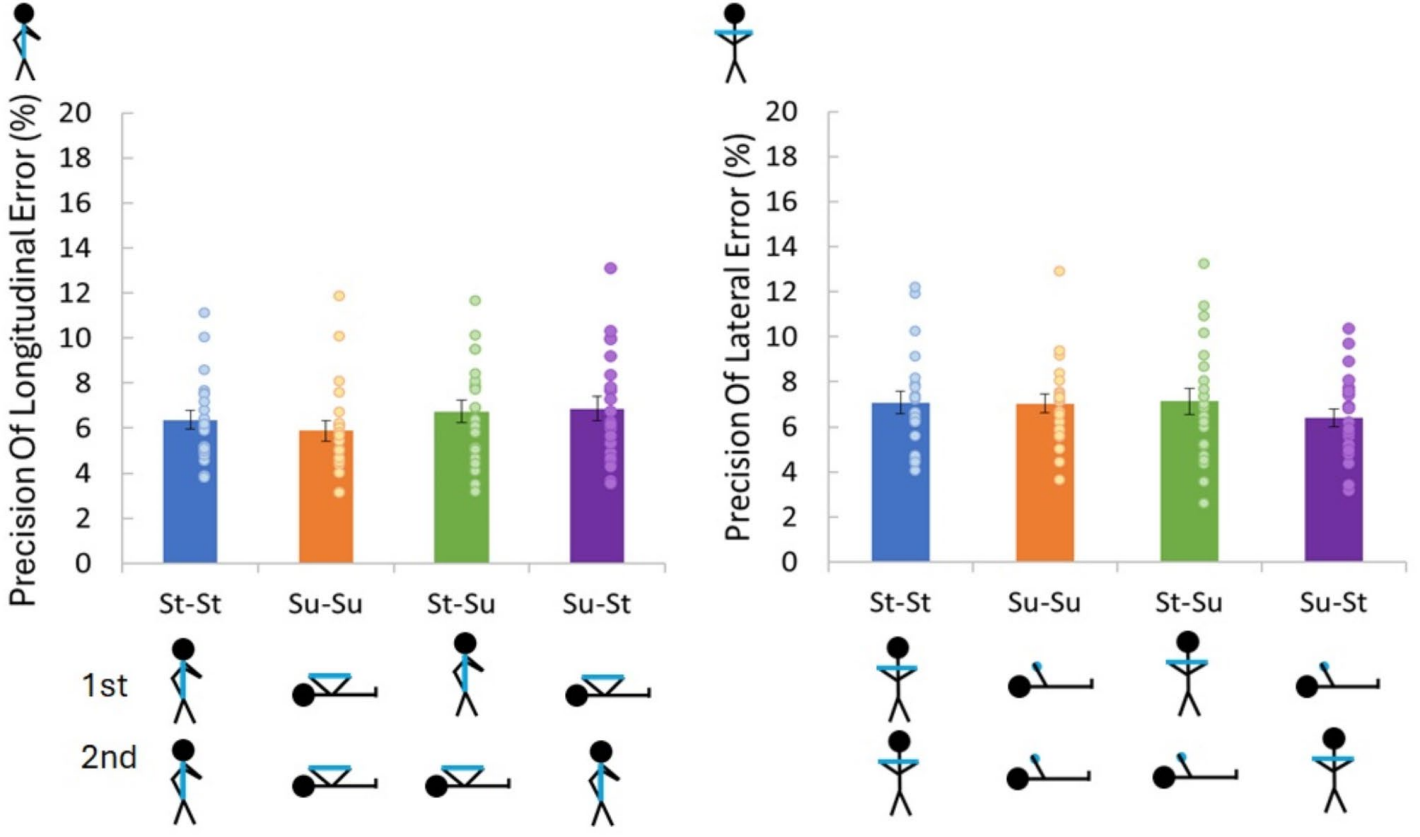
The precision of the adjusted length of the rod displayed as a percent error of the actual felt length (perceived size difference). The left panel shows the results for when the rod was held aligned longitudinally and the right panel for when the rod was held laterally. St–St, Standing–Standing posture; Su–Su, Supine–Supine posture; St–Su, Standing–Supine posture; Su–St, Supine–Standing posture. Error bars are ± standard error.

There was no significant effect of body orientation on the precision of the perceived size difference *F*(3, 63) = 0.91, *p* = 0.442, n_p_^2^ = 0.04 (see Fig. 3). Table 2 displays the post hoc tests along with the Bayes Factor for any comparison which failed to achieve significance.

**Table 2:** Pairwise comparisons for the precision for the different rod and body orientations.

Orientation	Condition (I)	Condition (J)	Perceived size difference (I-J)	Standard error (%)	Corrected alpha	95% confidence interval for difference (%)	Bayes factor 01
Longitudinally	St–St	Su–Su	0.482	0.515	1.000	− 1.018 to 1.981	4.044
	St–St	St–Su	− 0.365	0.573	1.000	− 2.033 to 1.303	5.040
	St–St	Su–St	− 0.491	0.619	1.000	− 2.292 to 1.311	4.538
	Su–Su	St–Su	− 0.847	0.643	1.000	− 2.718 to 1.024	2.731
	Su–Su	Su–St	− 0.972	0.643	0.872	− 2.844 to 0.899	2.141
	St–Su	Su–St	− 0.125	0.844	1.000	− 2.584 to 2.333	6.061
Laterally	St–St	Su–Su	0.052	0.688	1.000	− 1.950 to 2.054	6.109
	St–St	St–Su	− 0.051	0.625	1.000	− 1.872 to 1.770	6.106
	St–St	Su–St	0.692	0.591	1.000	− 1.028 to 2.412	3.220
	Su–Su	St–Su	− 0.103	0.558	1.000	− 1.729 to 1.524	6.027
	Su–Su	Su–St	0.640	0.588	1.000	− 1.074 to 2.353	3.510
	St–Su	Su–St	0.742	0.634	1.000	− 1.104 to 2.589	3.221

Asterisks indicate significant results. The alpha values have been corrected for the multiple comparisons (Bonferroni) such that a ‘corrected alpha’ of less than 0.05 indicates statistical significance. A Bayes factor (BF 01) above 1 indicates support for the null hypothesis and numbers below 1 indicate support for the test hypothesis.

### Lateral rod orientation

####  Measuring bias when the posture was not changed

When the participant maintained their posture and held the rod across the body there was no significant bias in the adjusted length of the rod while standing (St–St) *t*(21) = 0.629, *p* = 0.54, *d* = 0.13, but there was a significant bias while supine (Su–Su) *t*(21) = 3.50, *p* = 0.002, *d* = 0.75 where participants set it as larger than it really was (St–St: mean perceived size difference = 0.45%, SD = 3.39%; Su–Su: mean perceived size difference = 3.25%, SD = 4.36%). We found a Bayes Factor of BF = 5.067 for the St–St one sample t-test indicating moderate support for the null hypothesis.

#### Effect of posture on the perceived length of the rod

When the rod was held across the body, we observed a significant effect of body orientation, F(3, 63) = 4.99, *p* = 0.004, n_p_^2^ = 0.19 (Fig. 2) showing that the Su–Su condition differed from the St–St condition (Su–Su > St–St). No other effects were found. The St–Su condition had a mean perceived size difference of 0.80% (SD = 3.65%) while the Su–St condition had a mean perceived size difference of 3.63% (SD = 5.08%). We calculated the Bayes Factor for the any paired samples t-tests and provide the results alongside the results of frequentist analysis in Table 1 (the test hypothesis being that the two conditions differ from each other).

#### Effect of posture on the precision of the perceived length of the rod

There was no significant effect of body orientation on the precision of the perceived size differences, *F*(3, 63) = 0.64, *p* = 0.592, n_p_^2^ = 0.03 (see Fig. 3). Table 2 displays the post-hoc t-tests along with the Bayes Factor for each comparison.

## Discussion

This study investigated whether changes in posture influence perceived haptic length. When participants held a rod aligned with the long axis of their body there were systematic changes in the perceived length of the rod dependent on body posture. When participants changed their posture in between sampling and setting the length of a rod, we found a pattern of results consistent with an expansion of perceived haptic length while supine. This pattern of results was not found when the rod was held laterally. Our findings show that changes in posture not only influence the perceived visual length of a line^2,3^ but also the perceived haptic length with the results comparable to the findings of previous studies where a rod was used as a reference length to compare with a visual line^2,3^. Our results are consistent with the notion that the vestibular system encodes a representation of space with respect to gravity and that this representation might be distorted in different directions. Specifically, the vestibular system might provide a reference direction to which any such representation can be anchored, and that this representation is distorted.

See Table 3 for a breakdown of how our hypotheses fared.

**Table 3:** A list of our hypotheses (see Fig. 1) and whether they turned out to be true of false.

Hypothesis	Result	Support
General		
H1	General bias in setting rod size	TRUE
Is haptic size estimation independent of rod orientation?		
H2	No difference in precision between standing and supine in either rod orientation	FALSE
H3	No difference in precision between maintaining or changing posture for either rod orientation	FALSE
H4	Differences in perceived haptic size while supine depended on rod orientation	FALSE
Is haptic size estimation dependant on rod orientation?		
H5	Results consistent with haptic expansion while supine when moving from standing to supine when rod is held longitudinally	TRUE
H6	Results consistent with haptic expansion while supine when moving from supine to standing when rod is held longitudinally	TRUE
H7	When holding the rod laterally there is no effect of changing posture on perceived rod length	TRUE

As memory has been found to affect perceived size where we tend to remember objects as larger than they were^17^, we expected a general overestimation of the perceived size of the rod when participants felt the rod and then adjusted it to its remembered length 10s later without changing posture (Hypothesis 1). When participants held the rod longitudinally and maintained their posture there was indeed a bias to adjust the rod as longer compared to the actual length of the rod. If participants held the rod laterally and maintained their posture, a bias was only found when sample and setting were both done supine. When both tasks were done standing however, there was moderate evidence to support the null hypothesis that the condition did not differ from 0 (BF = 5.067). According to the idea that the size increases in memory^17^, this should also have been found in this condition. People have a lot of everyday experience holding objects in front of themselves with both hands while standing, such as trays, books, laptops, plates, etc. which might have contributed to their accurate performance. If the general bias to adjust objects as too large found in the current study, at least when the rod is held longitudinally, is due to a simple memory effect it is possible then that when the participants changed their posture the variations in size might reflect further distortions in memory. This could be due to an increase in executive costs from changing posture leading to a more rapid decay in memory. This implies that the size of the rod is felt accurately, just that the remembered size is altered. However, if the effect was indeed due to changes in the representation of the size of the object in memory, then the effect should be independent of rod orientation, which we did not observe in our study.

**Fig. 4:**
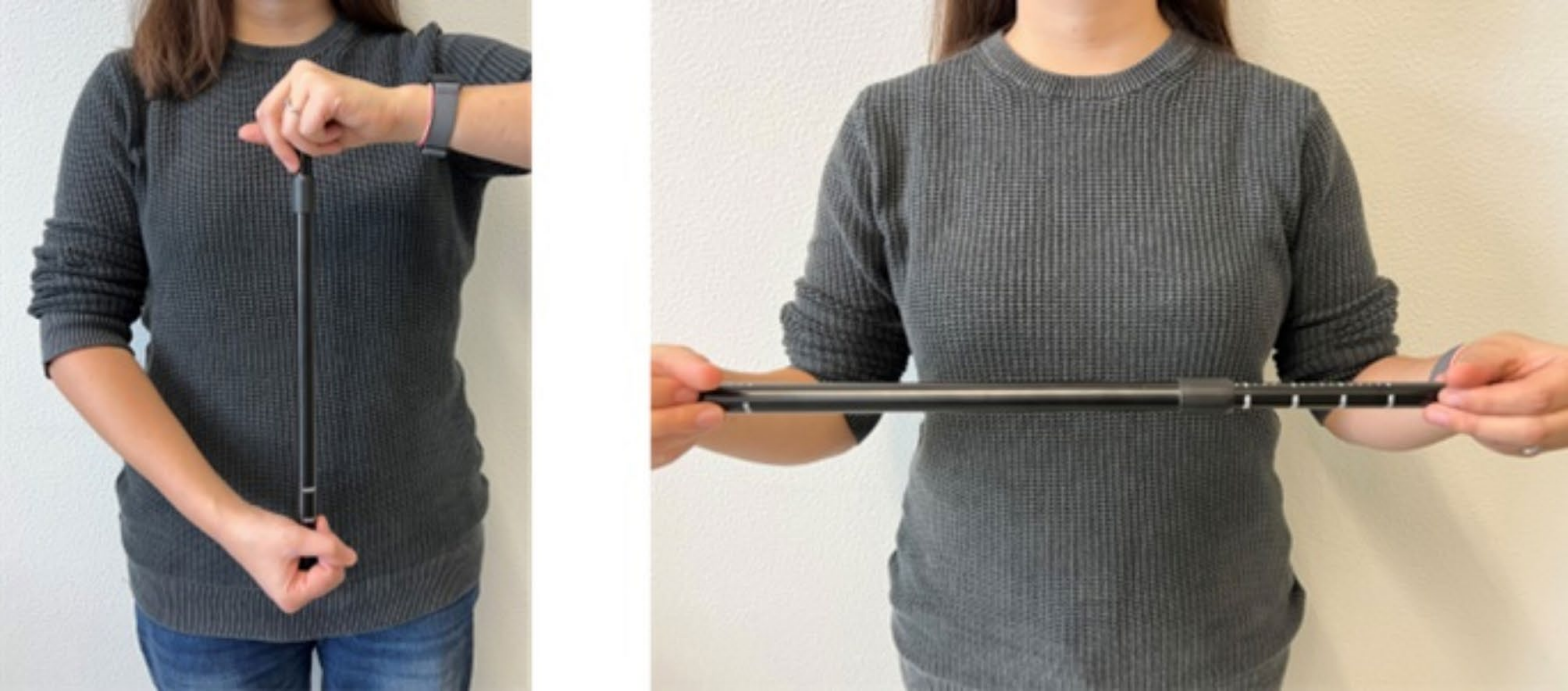
Photographs of the expandable rod used in the experiment. The left panel depicts the rod being held aligned with the long axis of the body; the right panel depicts it being held across the body.

We had hypothesized that if changes in perceived size during tilt were due to changes in the reliability of the vestibular cue with tilt, then there should be a decrease in the precision of the estimate of the size of the rod while supine compared to standing independent of rod orientation (Hypotheses 2). However, no such difference in precision was found, with typically moderate evidence to support the null hypothesis. Additionally, we had hypothesized that precision should be lower following a change in posture regardless of rod orientation (Hypothesis 3) however there were no changes in the precision of the size estimate for any posture combination or rod orientation (Figs. 3 and 4) which was also supported by generally moderate support for the null hypothesis for the individual comparisons. While some studies have found changes in the precision of estimates with body tilt^9,13,25^, others have not, both during microgravity exposure and body tilt^3,26^. Similar levels of precision in the different postures could indicate that the vestibular information is integrated similarly in the different postures. The lack of differences between the precisions of the estimates would seem to suggest that any effects found are unrelated to changes in the reliability of the vestibular cue during tilt.

The differences in the size estimation of the two rod orientations could be explained by the vestibular system being intimately involved in encoding a representation of the space around us^18,19^. Recent research has found modality-independent changes in our perception of the space around the body during parabolic flights and when the body is tilted^19^. For example, in a study by Morfoisse et al.^19^, participants were tasked with adjusting a rectangular prism so that it appeared as a cube while standing or supine, both visually and haptically. When the experimenters analyzed the adjusted sizes of the different sides of the cube with respect to participants’ body orientation no differences were found between postures. However, this result changed when they analyzed the adjusted cube size with respect to gravity. They found systematic differences in the adjusted size that were in opposite directions for the visual and haptic senses. They interpret their results as showing that the visual and haptic errors are related to the orientation of the cube with respect to the body and not to gravity. Our results however are consistent with the notion that the vestibular system encodes a representation of the space with respect to gravity which might help to explain Morfoisse et al.,’s^19 ^findings during parabolic flight where, during the microgravity phase both haptic and visual errors were affected in the same direction such that there was an increase in haptic overestimation and a decrease in visual underestimation. Additionally, such a haptic overestimation of the size of the rod in the current study could contribute to the explanation of previous findings of posture-related changes in visual size estimation^2,3^ and adds further evidence to the notion that the representation of space is distorted.

If the vestibular system were involved in encoding a representation of space with respect to gravity, we should observe changes in the perceived size of the rod as the posture was changed when the rod was held longitudinally but not when it was held laterally despite the participants being in the same posture (Hypotheses 5–7). In line with Hypotheses 5 and 6, we found that when the participants held the rod longitudinally and then changed their posture between sampling and setting, the adjusted lengths differed significantly by roughly 3–3.5% beyond what could be accounted for by the general memory-related bias found when they maintained their posture (see Fig. 2; magnitude of the averaged mean perceived size difference when posture is maintained—mean perceived size difference when posture is varied). Importantly, participants adjusted the rod significantly smaller following the St–Su posture change compared to the Su–St posture change. In line with previous results^2,3^, this pattern is consistent with a haptic expansion while supine such that if the participants felt the rod first while standing and then adjusted it while supine, the rod had to be adjusted smaller to match the previously felt size. If participants first felt the rod while supine and then moved to a standing posture, it had to be adjusted larger to match the previously felt size.

Conversely, when participants held the rod laterally and changed their posture, the estimates did not differ from when they maintained their posture (the adjusted lengths differed by roughly 1.1–1.8%) which was supported by the Bayes Factors showing either moderate support for the null hypothesis (St–St to St–Su and Su–Su to Su–St) or no evidence to support either hypothesis (St–St to Su–St). However, there was moderate support for the test hypothesis when comparing Su–Su to St–Su. Importantly, there was no difference between St–Su to Su–St and the Bayes Factor indicated no support for either mode, in line with Hypothesis 7. Our findings are supported by a recent paper^27^ that looked at the effect of body posture on the perceived length of a line/rod visually, haptically, and combined while standing, supine, and supine with arms bent 45°. In these different postures the head was kept at a fixed angle so just the body posture varied. Consistent with our findings when the rod was held laterally, they found no differences in perceived size between the different body postures. In our experiment the participants experienced the same posture combinations for both rod orientations however differences in perceived size only occurred when the orientation of the rod changed with respect to gravity. Additionally, the lack of difference between their supine posture and the supine posture with arms bent 45°^27^, would suggest that the results here are not due to differences in the proprioceptive cues when the arms are in the different postures.

Why might there be distortions in the perception of space respect to gravity? This remains an open question, but one reason could be due to the idea that when depth information regarding the ground surface is poor or missing, we perceive the ground surface to be sloped upwards^28,29^. This would lead to a misperception of object distance as closer than it really is, which would lead to misperceptions of size^28,29^. While this might help to explain the visual distortions during tilt^2,3^ it is less clear how this might apply to haptic stimuli. Another possible explanation could be due to the finding of an underconstancy during reaching when upright where there are distortions in perceived size/distance in the direction of reach space^30,31^. These studies have found that we misperceive the size/distance of objects close to us as being further away (or larger) towards the location of reach space, and objects further away from us as being closer (or smaller) to the location of reach space. If this representation of space is constructed based on a typical upright posture (in line with gravity) then when tilted the body might be perceived as further from this typical reach space thus exaggerating the underconstancy found.

## Conclusion

When participants were tilted from upright, the length of a rod they held between their hands was misperceived as longer than when they were upright but only if the rod’s orientation was changed with respect to gravity. If the rod did not change its orientation with respect to gravity (i.e., was held parallel to the axis of rotation) as the person was tilted, there was no effect on its perceived length. These observations are consistent with the idea that the vestibular system encodes a representation of space with respect to gravity. Such a haptic overestimation of the reference rod could contribute to the explanation for some previous findings on visual size estimation^2,3^ beyond the posture-related changes in visual size.

## Materials and methods

### Sample size estimation

To calculate the required sample size for this experiment we used G*Power (version 2.1.9.4) and set the statistical test as Repeated Measures within factors ANOVA and the type of analysis as the a priori sample size estimation. We calculated the expected effect size based on the partial eta squared, 0.514, from^2^ and set the effect size specification as “Cohen (1988) - Recommended”. The effect size was 1.03 with an alpha probability error of 0.05, a power of 0.95, the number of groups set to 1 and the number of measurements to 4 (corresponding to the different body posture combinations), and a non-sphericity correction of 1. This resulted in a minimum sample size of 18. To account for potential outliers, we increased the sample size to 22 participants.

### Participants

We recruited 22 participants (mean age = 25.94 years, SD = ± 3.36 years, 17 women, 5 men) from Justus Liebig University Giessen. Participants received compensation for participating at a rate of 8 euro/h or test subject’s hours. They reported no hearing or balance problems and had no current hand or arm injuries. Corrected visual impairment (glasses) was allowed, but potential participants with other vision problems were excluded. All participants provided written informed consent. The experiment was conducted in agreement with the principles of the Declaration of Helsinki (2013) and was approved by local Ethics Review Board at the Faculty of Psychology and Sports Science, Justus Liebig University Giessen, Germany (Certificate # 2019-0003). The experiment was performed in accordance with relevant guidelines and regulations.

### Apparatus and stimuli

#### Body postures

Participants either stood with their back against a wall (standing) or lay supine on a 71 cm x 195 cm x 65 cm massage bed with their feet against the wall. When completing the task, they were asked to keep their arms raised slightly off the bed/wall and their elbows and hands away from their body. This helped to keep proprioceptive information consistent across the two postures and to limit the participants’ ability to use their body as a spatial reference.

#### Haptic rod

The haptic rod was a black metallic telescopic rod made of two pieces that could vary between 32 cm and 64 cm (Fig. 4). The rod could be adjusted by applying pressure to the ends to shrink it or the ends could be pulled to lengthen it. The rod maintained a given length by means of a spring inside which hugged the walls. Participants were instructed to touch only the ends of the rod to prevent them from using their hands to estimate its length (see Fig. 4).

#### Procedure

After checking the exclusion criteria, the experimental instructions were explained. The participant was told in which orientation they would be holding the rod and in which of the two postures they would be tested first. Each posture was demonstrated to the participant. The participant was never shown the rod so that they could not get an estimate of its visual size.

The participant adopted the first posture (standing or supine) and put on the blindfold. The experimenter then set the rod to one of three lengths (40, 46, 52 cm) using a tape measure. The participant felt the presented rod for 5 s, then the experimenter took the rod from them. The participant then either maintained (standing–standing, supine–supine) their posture for 10 s, or they had 10 s to change their posture (standing–supine, supine–standing). If the participant finished changing postures faster than 10 s they simply waited in the new posture for the remainder of the 10 s. During the 10 s the experimenter set the rod to a random length between 38 and 58 cm using the tape measure. The random lengths were determined using the RandBetween() function in Excel (version 2304). When not holding the rod participants were instructed to keep their hands at their sides.

After 10 s, the rod was handed back to the participant and they adjusted it to its previously felt length at their own pace. When they were satisfied, the experimenter took the rod back and measured and recorded its length. The rod was then set to the next test length (40, 46, or 52 cm) while the participant changed their posture or maintained their posture for the next trial. Each of the three lengths was repeated three times for a given posture combination (for a total of nine repeats for each posture combination). The order of the presentation of the lengths was randomized for all conditions and participants.

After completing all nine trials in a given posture combination, the participant was informed about the next posture combination. After completing all four posture combinations (standing–standing, standing–supine, supine–supine, supine–standing) for one rod orientation (9 × 4 = 36 trials) the participant removed the blindfold, and the experimenter instructed the participant on the new rod orientation (either aligned with the long axis of the body or held across the body) without them seeing the rod. The experiment then proceeded as with the first rod orientation. The order of the posture combinations was randomly determined across participants and rod orientation and the order of the rod orientations was counterbalanced between participants.

This created a total of 4 posture combinations (Standing–Standing, Supine–Supine, standing–supine, and Supine–Standing) and 2 rod orientation (along the long axis of the body or across the chest). With 9 trials per posture combination for a total of 72 trials.

The total duration of the experiment was approximately 2 h.

### Data analysis

#### Measuring bias when the posture was not changed

In order to test for any biases when setting the size of the rod (Hypothesis 1), we employed two sets of one sample, 2-tailed, t-tests to check if the difference in the standing–standing and supine–supine adjusted rod lengths differed from 0. This was done for both orientations of the rod.

#### Affect of posture on perceived size

In order to determine if body orientation influenced perceived haptic size, we analyzed the adjusted length of the rod using IBM SPSS Statistics (Version 27, IBM Cooperation, 2020). For each trial, we calculated the difference between the adjusted length of the rod and the initial length of the rod. This error value was then divided by the initial length of the rod and multiplied by 100 to get the percent error. We then averaged the percent error for each posture combination and rod orientation for each participant. We will refer to this as the “perceived size difference” as it is the difference between the adjusted and initial rod length. A positive number indicates that the participant adjusted the length of the rod as longer than the initial length and a negative number indicates that the adjusted length of the rod was shorter than its initial length.

To assess possible effects of body orientation on haptic size perception of the rod in all posture combinations, we employed two separate repeated measures ANOVAs: one for each rod orientation. This was done to test Hypotheses 1, 4, 5, 6, and 7. The tests of the ANOVAs’ assumptions revealed that the residuals of the data appeared normally distributed with no significant outliers. A significant outlier was determined as any point that fell outside of the 1st or 3rd interquartile range by 3 times the interquartile range. Sphericity can be assumed (*p* > 0.05). Multiple comparisons were corrected with the Bonferroni correction.

To confirm our null findings, we followed up the frequentist analysis by calculating the inverse Bayes Factor (BF01) for each test. The default settings were used and the priors were based on the variance with a diffuse setting. BF01 is evidence for the null hypothesis (H0) over the test hypothesis (H1),P(D|H0). With the BF01 Bayesian analysis, a Bayes factor above 1 indicates support for H0 and numbers below 1 indicate support for H1. Values between 3 and 10 or 0.3 and 0.1 indicate moderate evidence, values greater than 10 or less than 0.10 indicate strong support, and values between 0.3 and 1–3 indicate no evidence (anecdotal) for or against the null hypothesis (see^32^ Table 1, where M_j_ would be the null hypothesis and M_i_ would be the testing hypothesis).

#### Affect of posture on the precision of perceived size

For each participant we calculated the standard deviation of the perceived size difference within a posture combination for each rod orientation. The reciprocal of this value can be taken as the precision of the estimate. In the current paper we report the standard deviation that increases when precision decreases. To assess possible effects of body orientation on the precision of the haptic size perception of the rod in all posture conditions, we employed two separate repeated measures ANOVAs: one for each rod orientation. This tested Hypotheses 2 and 3. The assumptions of the ANOVAs were tested, and the residuals of the data appeared normally distributed. There was one significant outlier in each of the repeated measures ANOVAs which fell outside of the 3rd interquartile range by 3 times the interquartile range. Removing these outliers did not affect the results qualitatively so the data points were left in. Sphericity can be assumed (*p* > 0.05). Multiple comparisons were corrected with the Bonferroni correction. To confirm null findings, we calculated the inverse Bayes Factor (BF01) for each test (see above).

## Data Availability

The data collected for this work are publicly available at the Open Science Framework: 10.17605/OSF.IO/G2ZVB.
